# Volasertib as a monotherapy or in combination with azacitidine in patients with myelodysplastic syndrome, chronic myelomonocytic leukemia, or acute myeloid leukemia: summary of three phase I studies

**DOI:** 10.1186/s12885-022-09622-0

**Published:** 2022-05-21

**Authors:** Uwe Platzbecker, Joerg Chromik, Jan Krönke, Hiroshi Handa, Stephen Strickland, Yasushi Miyazaki, Martin Wermke, Wataru Sakamoto, Yoshifumi Tachibana, Tillmann Taube, Ulrich Germing

**Affiliations:** 1 Medical Clinic and Policlinic I, Hematology and Cellular Therapy, University Hospital Leipzig, Johannisallee 32, D-04103 Leipzig, Germany; 2 Department of Hematology and Medical Oncology, University Hospital Frankfurt, Frankfurt, Germany; 3 Department of Internal Medicine, University Hospital of Ulm, Ulm, Germany; 4 Department of Hematology, Gunma University Graduate School of Medicine, Maebashi, Japan; 5 Division of Hematology and Oncology, Vanderbilt-Ingram Cancer Center, Nashville, TN USA; 6 Department of Hematology, Nagasaki University Hospital, Nagasaki City, Japan; 7 NCT/UCC Early Clinical Trial Unit, Technical University Dresden, University Hospital Carl Gustav Carus, Dresden, Germany; 8 Biostatistics and Data Science Japan, Medical Division, Nippon Boehringer Ingelheim, Tokyo, Japan; 9 Clinical Operations Japan, Nippon Boehringer Ingelheim, Tokyo, Japan; 10 Therapeutic Area Oncology Medicine, Boehringer Ingelheim International, Biberach, Germany; 11 Department of Hematology, Oncology and Clinical Immunology, Heinrich-Heine University, Dusseldorf, Germany

**Keywords:** Volasertib, Phase I, Myelodysplastic syndrome, Chronic myelomonocytic leukemia, Acute myeloid leukemia

## Abstract

**Background:**

This report summarizes three phase I studies evaluating volasertib, a polo-like kinase inhibitor, plus azacitidine in adults with myelodysplastic syndromes (MDS), chronic myelomonocytic leukemia, or acute myeloid leukemia.

**Methods:**

Patients received intravenous volasertib in 28-day cycles (dose-escalation schedules). In Part 1 of 1230.33 (Study 1; NCT01957644), patients received 250–350 mg volasertib on day (D)1 and D15; in Part 2, patients received different schedules [A, D1: 170 mg/m^2^; B, D7: 170 mg/m^2^; C, D1 and D7: 110 mg/m^2^]. In 1230.35 (Study 2; NCT02201329), patients received 200–300 mg volasertib on D1 and D15. In 1230.43 (Study 3; NCT02721875), patients received 110 mg/m^2^ volasertib on D1 and D8. All patients in Studies 1 and 2, and approximately half of the patients in Study 3, were scheduled to receive subcutaneous azacitidine 75 mg/m^2^ on D1–7.

**Results:**

Overall, 22 patients were treated (17 with MDS; 12 previously untreated). Across Studies 1 and 2 (*n* = 21), the most common drug-related adverse events were hematological (thrombocytopenia [*n* = 11]; neutropenia [*n* = 8]). All dose-limiting toxicities were grade 4 thrombocytopenia. The only treated patient in Study 3 experienced 18 adverse events following volasertib monotherapy. Studies 1 and 2 showed preliminary activity (objective response rates: 25 and 40%).

**Conclusions:**

The safety of volasertib with azacitidine in patients with MDS was consistent with other volasertib studies. All studies were terminated prematurely following the discontinuation of volasertib for non-clinical reasons by Boehringer Ingelheim; however, safety information on volasertib plus azacitidine are of interest for future studies in other diseases.

## Introduction

Myelodysplastic syndromes (MDS), chronic myelomonocytic leukemias (CMML), and acute myeloid leukemias (AML) are related myeloid malignancies characterized by hematopoietic insufficiency, affecting clonal hematopoietic stem cells. Patients with MDS, CMML, and AML can receive risk-adapted treatment, including curative hematological stem cell transplantation (HSCT), palliative hypomethylating agents, and chemotherapy [1–3].

Volasertib is a potent, selective, small molecule inhibitor of Polo-like kinases (PLK) PLK1, PLK2, and PLK3 [4]. PLK1, a member of the PLK family, controls essential steps during mitosis and its activity peaks during late G2 and M phases. It is highly expressed in proliferating cells, and is overexpressed in a broad spectrum of cancers, making it a potential target for anti-cancer therapy [4].

Hypomethylating agents, such as azacitidine and decitabine, are potent inhibitors of DNA methyltransferase that function to restore normal cell growth and differentiation [5]. These therapies are recommended for patients with MDS and AML who are not suitable for HSCT [6, 7]. A randomized phase III trial in patients with AML (*n* = 488) showed that azacitidine improved overall survival and was more effective than conventional care regimens [8]. However, positive outcomes with azacitidine remain limited and short-lived in the majority of patients [7]. There are virtually no predictive parameters for response to hypomethylating agents, and the treatment response rate is approximately 50% [7, 9]. Therefore, there is a strong medical need for the development of novel therapies.

The combination of a drug that interferes with cell cycle progression or S phase (e.g., azacitidine) with volasertib might be expected to produce additive or synergistic effects. The additive activity of the combination of volasertib and azacitidine was confirmed in preclinical studies in AML cells [10].

Volasertib showed clinical activity in a phase I/II study in adult patients with relapsed/refractory AML [11], and in combination with low-dose cytarabine (LDAC) in adult patients with AML unsuitable for intensive treatment [12]. However, a phase III study (POLO-AML-2; NCT01721876), conducted to confirm the promising phase I/II results, evaluating volasertib and LDAC in previously untreated patients aged ≥ 65 years with AML who were ineligible for intensive therapy, did not meet its primary endpoint [13].

Combining volasertib with an agent that has a different mechanism of action, such as azacitidine, was therefore considered a rational treatment approach for evaluation in the treatment of patients with MDS, AML, and CMML.

This report summarizes the design and results of three phase I studies that were conducted to determine the safety, tolerability, and preliminary anti-cancer activity of volasertib monotherapy and in combination with azacitidine in adults with MDS, CMML, or AML. Although the global clinical development of volasertib by Boehringer Ingelheim was discontinued for non-clinical reasons in December 2016 while these studies were ongoing, published information on the safety of volasertib in combination with azacitidine in this patient population may be of scientific interest [14]. Of note, the current licensing agreement is not limited to clinical development to paediatrics, and development in adults might be considered in the future.

## Methods

### Study design and patients

This report covers three open-label, phase I, dose-escalation studies investigating volasertib monotherapy and combination therapy with azacitidine in adult patients with MDS, AML, and/or CMML: studies 1230.33 (Study 1), 1230.35 (Study 2), and 1230.43 (Study 3).

Inclusion criteria differed across the three studies in terms of treatment history. Study 1 (NCT01957644) investigated the combination of volasertib and azacitidine in patients with previously untreated intermediate-2 or higher-risk MDS or CMML, who were not candidates for HSCT. Study 2 (NCT02201329) investigated the combination in Japanese patients with intermediate-2 or higher-risk MDS or CMML, who were also not candidates for HSCT; patients could have previously received treatment with azacitidine or be untreated. Study 3 (NCT02721875) investigated volasertib monotherapy or volasertib plus azacitidine in patients with higher-risk MDS, CMML, or AML after failure of treatment with hypomethylating agents. Other inclusion criteria included: age ≥ 18 years (Studies 1 and 3) or between 20 and 80 (Study 2), Eastern Cooperative Oncology Group performance status (ECOG PS) 0/1 (Study 2), or ≤ 2 (Studies 1 and 3).

Patients were excluded from all three studies if they had received prior treatment with volasertib or had a concomitant malignancy requiring active therapy. Patients in Study 1 were excluded if they had received prior or concomitant therapy for higher-risk MDS or prior treatment with any PLK1 inhibitor. In Study 2, patients were excluded if they had received systemic therapy for MDS within 14 days before treatment. Patients were excluded in Study 3 if they had received any prior systemic therapy for MDS, CMML, or AML within 14 days of treatment, and if they had received any prior PLK inhibitor or hypomethylating treatment.

**Fig. 1:**
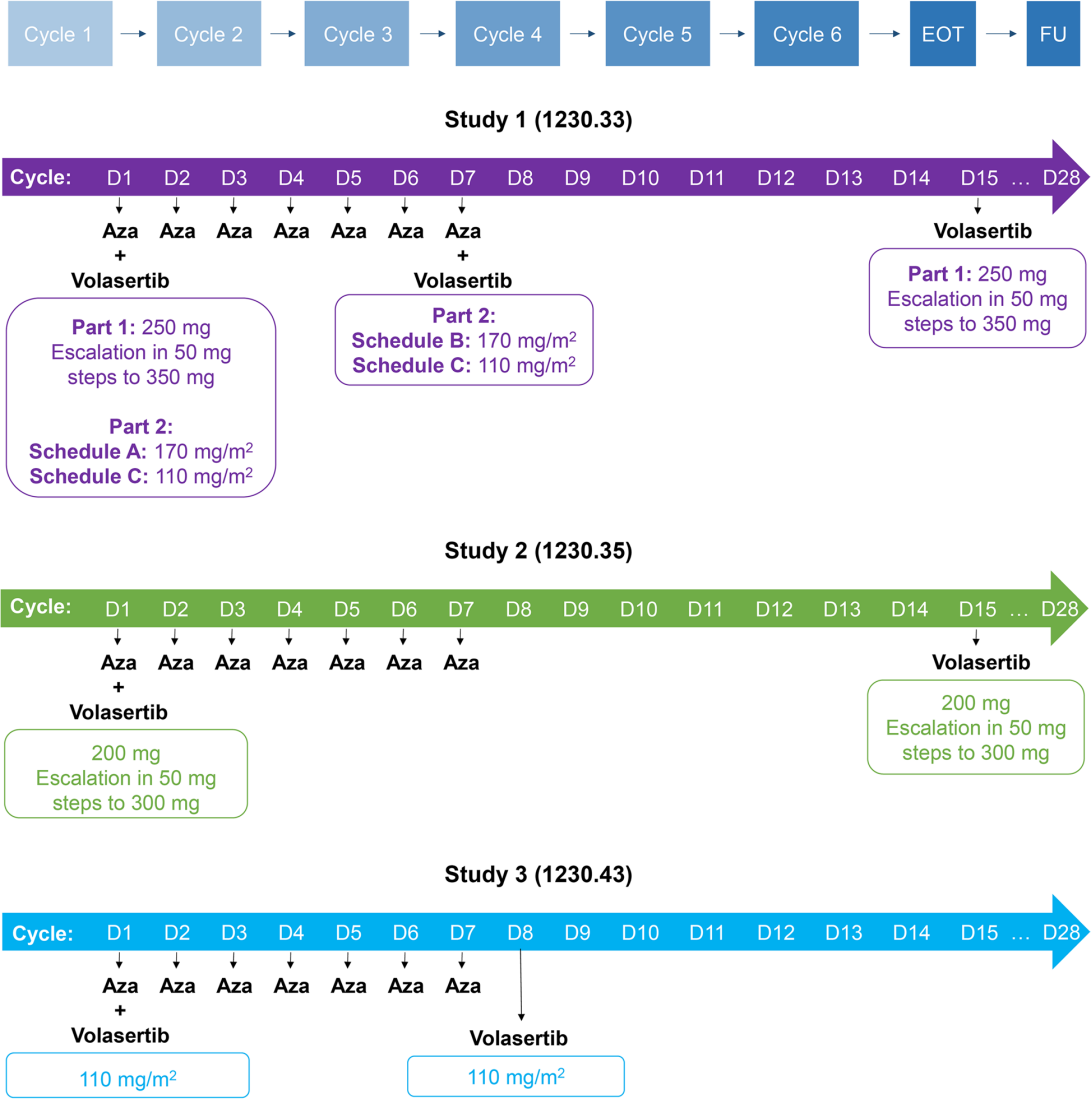
Overview of study design and treatment schedule in Studies 1, 2, and 3. Azacitidine was given at 75 mg/m^2^ to all patients in Studies 1 and 2, and approximately half of patients in Study 3. All planned cycles were 28 days. *Aza*, azacitidine; *D*, day; *EOT*, end of treatment; *FU*, follow-up

In all three studies, escalating doses of intravenous volasertib were administered to determine the maximum tolerated doses (MTD); all cycles were 28 days. In Part 1 of Study 1, patients received volasertib infusions on days 1 and 15, starting at a flat dose of 250 mg and escalating in 50 mg steps to 350 mg (Fig. 1). In Part 2 of Study 1, patients received body surface area (BSA)-adapted doses of volasertib and different schedules (Schedule A [planned], volasertib 170 mg/m^2^ on day 1; Schedule B, volasertib 170 mg/m^2^ on day 7; Schedule C, volasertib 110 mg/m^2^ on days 1 and 7). After the MTD was determined, it was planned to enroll up to 40 patients in an expansion cohort to characterize safety further.

In Study 2, a schedule of infusions on days 1 and 15 was planned, starting at a flat dose of 200 mg in Cohort 1 and escalating in 50 mg steps to 300 mg (250 mg in Cohort 2 and 300 mg in Cohort 3). In Study 3, a schedule of infusions on days 1 and 8 was planned, starting at a dose of 110 mg/m^2^ (other doses and another schedule were also planned).

Patients in Studies 1 and 2 also received subcutaneous azacitidine 75 mg/m^2^ on days 1–7 of the 28-day cycle. Study 3 planned to evaluate two different treatment schedules: volasertib monotherapy (Schedule A) and subcutaneous or intravenous azacitidine in combination with volasertib (Schedule B). The dose escalation cohorts for each Schedule were planned to be enrolled alternately.

All trials were carried out in accordance with the principles of the Declaration of Helsinki, with the International Conference on Harmonization Harmonised Tripartite Guideline for Good Clinical Practice (GCP), and with applicable regulatory requirements. All patients provided written informed consent.

### Endpoints

The primary objective of each study was to determine the MTD of volasertib in combination with azacitidine (or as monotherapy in Study 3), which was defined as the dose level in which less than 2 of 6 patients (Study 1), not more than 1 of 6 patients (Study 2), or not more than 33% of patients (Study 3) experienced a dose-limiting toxicity (DLT).

DLTs were defined as any of the following considered to be drug-related: 1) grade ≥ 3 (Common Terminology Criteria for Adverse Events [CTCAE] Version 3.0 or 4.03) non-hematological toxicity (exceptions were untreated nausea, vomiting, or diarrhea; clinically non-significant laboratory abnormalities, or laboratory abnormalities that resolved spontaneously; febrile neutropenia or grade 3 infection if the patient recovered with appropriate treatment within 7 days [all studies]); 2) inability to deliver the full dose of volasertib according to the assigned dose level within cycle 1; 3) treatment delay of ≥ 4 weeks due to drug-related adverse events (DRAEs).

Further safety endpoints included the incidence and intensity of adverse events (AEs) graded according to CTCAE Version 3.0 or Version 4.03. Efficacy was a secondary endpoint in these studies and was assessed by determining objective response to treatment according to International Working Group 2006 criteria [15]. In Studies 1 and 3, the objective response rate was calculated (proportion of patients with complete remission [CR] or partial remission [PR]).

### Statistical analyses

All analyses were summarized descriptively; no formal hypothesis testing was planned.

### Protocol amendments

#### Study 1

Following reports of an increased rate of fatal infection in the phase III 1230.14 trial of volasertib in patients with AML, Study 1 was modified: volasertib dosing adaptation to the patient’s individual BSA instead of a volasertib flat starting dose of 250 mg and the introduction of volasertib dosing schedules A (day 1), B (day 7), and C (days 1 and 7) instead of a single dosing schedule (days 1 and 15).

#### Study 2

In consideration of the DLT occurrence status in Study 1, planned doses in Cohorts 2 and 3 were changed from 300 mg to 250 mg for Cohort 2 and from 350 mg to 300 mg for Cohort 3, with the change of number of patients per cohort from 3-6 to 6, and Cohort Intermediate was deleted.

#### Study 3

The amendments to the protocol for Study 3 were as follows: the analysis of efficacy was to be performed only for the secondary endpoint because only 1 patient was treated in this study.

## Results

### Conduct of studies, patients and exposure

Demographic and baseline characteristics were similar across these three studies (Table 1), with a trend for a smaller BSA in Japanese patients (Studies 2 and 3). All patients were white in Study 1 and all patients were of Asian ethnic origin (Japanese) in Studies 2 and 3.

**Table 1:** Baseline and demographic characteristics

	Study 1 Part 1 (*n* = 13)	Study 1 Part 2 (*n* = 3)	Study 2 (*n* = 5)	Study 3 (*n* = 1)
Male/Female, n (%)	11 (85)/2 (15)	2 (67)/1 (33)	3 (60)/2 (40)	1 (100)/0
Mean age, years (SD)	68.6 (7.7)	N/A	73.2 (4.6)	69 (N/A)
ECOG PS, n (%)		N/A		
0	4 (31)		4 (80)	0
1	7 (54)		1 (20)	1 (100)
2	2 (15)		0	0
Disease, n (%)		N/A		
MDS	12 (92)		5 (100)	0
CMML	1 (8)		0	0
AML	0		0	1 (100)
IPSS classification at screening, n (%)		N/A		
Int-2 (1.5–2.0)	9 (69)		2 (40)	0
High (≥ 2.5)	4 (31)		3 (60)	1 (100)
Previous treatments, n (%)		N/A		
No prior treatment	11 (85)		1 (20)	0
Azacitidine	0		4 (80)	1 (100)
Cytarabine	0		1 (20)	0
Aclarubicin hydrochloride	0		1 (20)	0
Growth factor	1 (8)		0	0
Lenalidomide	1 (8)		0	0
Other	2 (15)		0	0
Median BSA, m^2^ (range)	1.9 (1.7–2.2)	N/A	1.6 (1.4–1.8)	1.7 (N/A)
Median body weight, kg (range)	79.9 (64.2–99.2)	N/A	59.5 (48.0–69.0)	60.5 (N/A)

*AML*, acute myeloid leukemia; *BSA,* body surface area; *CMML*, chronic myelomonocytic leukemia; *ECOG PS*, Eastern Cooperative Oncology Group performance status; *IPSS*, international prognostic scoring system; *MDS*, myelodysplastic syndrome; *N/A*, data not available; *SD*, standard deviation

In Study 1, 16 patients were treated. In Part 1, 7 patients received volasertib 250 mg plus azacitidine, and 6 patients received volasertib 300 mg plus azacitidine. The median treatment duration was 117 days (range, 2–393 days) and the median number of cycles initiated was 4 cycles (range, 1–10 cycles). In Part 2, 2 patients received Schedule B (volasertib 170 mg/m^2^ on day 7 and azacitidine 75 mg/m^2^ on days 1–7). These patients received treatment for 7 and 224 days, and started 1 and 5 cycles, respectively. One patient received Schedule C in Part 2 (volasertib 110 mg/m^2^ on days 1 and 7 and azacitidine 75 mg/m^2^ on days 1–7). This patient received treatment for 7 days and initiated 1 cycle. The study was stopped after these 16 patients were treated due to discontinuation of the development of volasertib for non-clinical reasons.

In Study 2, 5 Japanese patients were treated with volasertib 200 mg plus azacitidine. The median treatment duration was 108 days (range, 15–324 days). The median number of cycles initiated was 4 cycles (range, 1–8 days). Enrollment was stopped because DLT events (both thrombocytopenia) were reported in 2 patients, and the condition to escalate to the next dose level was not met.

In Study 3, 1 patient was treated with volasertib 110 mg/m^2^ monotherapy but withdrew from the study because of progressive disease (PD). The patient initiated 2 cycles and received four administrations of volasertib. The study was then stopped due to discontinuation of the development of volasertib.

### Safety

AEs were reported in all treatment groups across the three studies. The most common AEs were thrombocytopenia and neutropenia (Tables 2 and 3). DLTs reported for volasertib in combination with azacitidine were all grade 4 thrombocytopenia.

**Table 2:** Summary of AEs in Studies 1 (Part 1) and 2 in patients receiving volasertib and azacitidine

*n* (%)	Study 1 Part 1 (*n* = 13)	Study 2 (*n* = 5)
Any AE	12 (92)	5 (100)
DRAE	12 (92)	5 (100)
AE leading to dose reduction of volasertib	3 (23)	1 (20)
AE leading to dose reduction of azacitidine	0	2 (40)
AE leading to discontinuation of trial medication	6 (46)	0
SAE	8 (62)	2 (40)
DLT	2 (15)	2 (40)
Worst CTCAE grade		
Grade 1	0	0
Grade 2	0	0
Grade 3	2 (15)	0
Grade 4	10 (77)	5 (100)
Grade 5	0	0

*AE* adverse event, *CTCAE* Common Terminology Criteria for Adverse Events, *DLT* dose-limiting toxicity, *DRAE* drug-related adverse event, *SAE* serious adverse event

**Table 3:** Most common adverse events occurring in ≥ 30% of patients receiving volasertib and azacitidine in Studies 1 (Part 1) and 2

*n* (%)	Study 1 Part 1 (*n* = 13)					Study 2 (*n* = 5)				
	All	G1	G2	G3	G4	All	G1	G2	G3	G4
Injection site reaction	0	0	0	0	0	5 (100)	4 (80)	1 (20)	0	0
Thrombocytopenia	10 (77)	0	0	2 (15)	8 (62)	2 (40)	0	0	2 (40)	0
Neutropenia	7 (54)	0	1 (8)	0	6 (46)	1 (20)	0	0	0	1 (20)
Diarrhea	6 (46)	5 (39)	0	1 (8)	0	2 (40)	2 (40)	0	0	0
Decreased appetite	6 (46)	4 (31)	2 (15)	0	0	1 (20)	0	1 (20)	0	0
Rash	6 (46)	5 (39)	1 (8)	0	0	0	0	0	0	0
Febrile neutropenia	2 (15)	0	0	2 (15)	0	2 (40)	0	0	2 (40)	0
Pneumonia	3 (23)	0	1 (8)	2 (15)	0	2 (40)	0	1 (20	1 (20)	0
Pyrexia	3 (23)	3 (23)	0	0	0	2 (40)	2 (40)	0	0	0
Pharyngitis	0	0	0	0	0	2 (40)	2 (40)	0	0	0
White blood cell count decreased	0	0	0	0	0	2 (40)	0	0	1 (20)	1 (20)
Hypersensitivity	0	0	0	0	0	2 (40)	1 (20)	1 (20)	0	0
Fatigue	5 (39)	2 (15)	3 (23)	0	0	1 (20)	1 (20)	0	0	0
Constipation	5 (39)	4 (31)	0	1 (8)	0	1 (20)	0	1 (20)	0	0
Cough	5 (39)	5 (39)	0	0	0	0	0	0	0	0
Nausea	5 (38)	4 (31)	1 (8)	0	0	0	0	0	0	0
Blood creatinine increased	4 (31)	2 (15)	2 (15)	0	0	0	1 (20)	1 (20)	0	0
Anemia	4 (31)	0	3 (23)	1 (8)	0	0	1 (20)	0	0	1 (20)
Vomiting	4 (31)	3 (23)	1 (8)	0	0	0	0	0	0	0
Erythema	4 (31)	3 (23)	1 (8)	0	0	0	0	0	0	0
Alopecia	4 (31)	4 (31)	0	0	0	0	0	0	0	0

*G* grade

#### Study 1

**Fig. 2:**
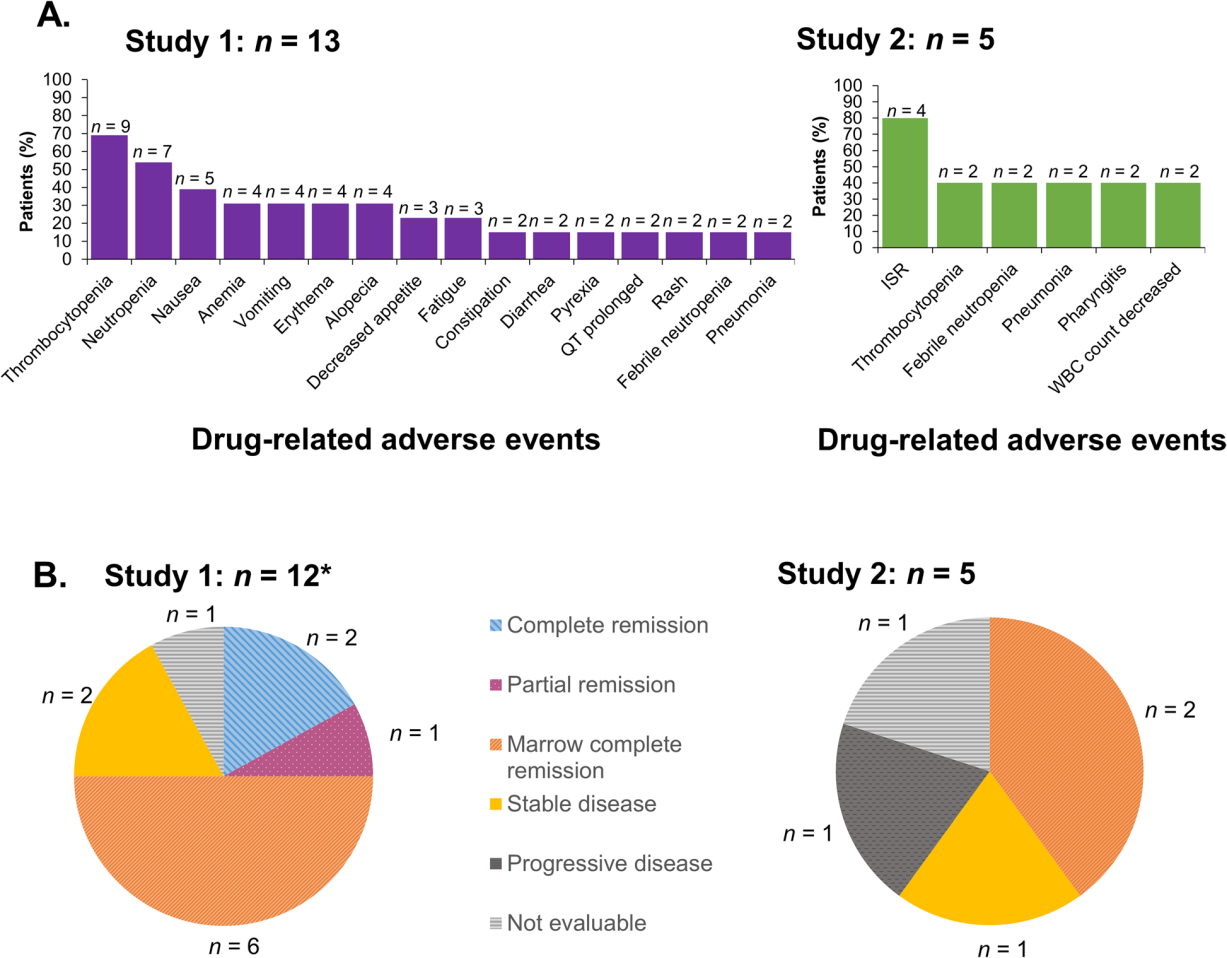
**a** Drug related adverse events (%) reported in ≥ 2 patients and **b** response in Study 1 (Part 1; *n* = 13) and Study 2 (*n* = 5). *One patient was not included in the efficacy evaluation in Study 1.  ISR, injection site reaction; *WBC,* white blood cell

In all treatment cycles in Part 1, at least one AE was reported for 12 of 13 patients. The most common AEs were thrombocytopenia (10 patients; 77%) and neutropenia (7 patients; 54%; Table 3). Over all treatment cycles, 10 patients (77%) had at least one grade 4 AE and 2 patients (15%) had at least one grade 3 AE. Three patients (23%) permanently reduced the volasertib dose due to AEs. Eight patients (62%) reported serious AEs (SAEs; Table 2); 2 patients had febrile neutropenia which was classed as an SAE, and 2 patients had pneumonia; the other SAEs were each reported in 1 patient only. Thrombocytopenia (9 patients; 69%), neutropenia (7 patients; 54%), and nausea (5 patients; 38%) were the most common DRAEs (Fig. 2a). In Part 2, the patient on Schedule C had SAEs (sepsis, hypokalemia, and febrile neutropenia). Two DLTs were reported and both were grade 4 thrombocytopenia; one in the volasertib 250 mg cohort, and one in the volasertib 300 mg cohort. As the study was discontinued, the MTD of volasertib in combination with azacitidine could not be determined.

#### Study 2

In all treatment cycles, at least one AE was reported for all 5 patients (Table 3) and all patients experienced at least one drug-related AE (Table 2). All patients experienced at least one grade 4 AE. No patients experienced AEs leading to discontinuation of study medication. AEs leading to dose reduction of volasertib and azacitidine were reported for 1 and 2 patients, respectively (Table 2). Two of 5 patients in the 200 mg cohort experienced DLTs, both grade 4 thrombocytopenia; hence, the MTD of volasertib on days 1 and 15 of a 28-day cycle in combination with azacitidine for Japanese patients was considered to be < 200 mg. Injection site reaction was the most common DRAE (Fig. 2a).

#### Study 3

The patient with AML who received volasertib monotherapy experienced 18 AEs, none of which were considered by the investigator to be drug-related. One AE was an SAE, grade 3 lower gastrointestinal hemorrhage, which required prolongation of hospitalization. Other grade 3 AEs were injection site infection, pneumonia, and cataracts.

### Efficacy

Preliminary signs of clinical activity were observed in Studies 1 and 2.

#### Study 1

In Part 1 of Study 1, 2 of the 12 evaluable patients (17%) had CR and 1 (8%) had PR, 6 patients (50%) had marrow CR (mCR), 2 patients (17%) had stable disease (SD), and 1 patient was not evaluable (Fig. 2b). In Part 2, none of the patients had an objective response. One patient had mCR and 2 were not evaluable.

#### Study 2

In Study 2, 2 of the 5 evaluable patients (40%) achieved mCR. One patient (20%) had SD; 1 patient (20%) had PD, and 1 patient (20%) was not evaluable (Fig. 2b). Response durations in the 2 patients with mCR were 221 days and 157 days.

#### Study 3

In Study 3, the patient experienced PD after Cycle 2; no CR, PR, nor hematological improvement were observed during Cycle 1 or 2.

## Discussion

These three studies were all prematurely discontinued when the clinical development of volasertib was discontinued, following a strategic decision by the sponsor; however, they provide useful insights into the safety profile and preliminary clinical activity of a PLK inhibitor as a treatment for MDS/CMML.

The safety profile of volasertib monotherapy or in combination with azacitidine was assessed in all three studies. All DLTs reported for volasertib in combination with azacitidine were grade 4 thrombocytopenia.

Volasertib, administered as a flat-dosing schedule (250 mg and 300 mg), in combination with azacitidine had a clinically manageable safety profile in Study 1. However, the MTD of volasertib in combination with azacitidine was not determined as the study was discontinued, meaning that only limited data are available on the tolerability of the combination regimen in this study. Of note, Study 1 had a protocol deviation from the 3 + 3 design. Of the 7 patients who received the 250 mg dosage, 6 were in the escalation phase, and 1 was in expansion phase. Initially, two DLTs were reported at 300 mg and the MTD was determined as 250 mg. Per protocol, recruitment to the MTD extension cohort (250 mg) was opened and 1 patient was enrolled. The study was then modified and Part 2 (BSA-adapted dosing) started. According to the final analysis, the previously concluded MTD of volasertib 250 mg in combination with azacitidine is not valid and it was concluded that one of the two DLTs in the 300 mg cohort did not meet the DLT criteria. Therefore, one DLT was reported in the volasertib 250 mg plus azacitidine cohort, and one was reported in the volasertib 300 mg plus azacitidine dose cohort in the final analysis of Part 1 (i.e. no MTD determined in Part 1).

In Study 2, the starting flat dose of volasertib 200 mg in combination with azacitidine exceeded the MTD in Japanese patients, and the study was discontinued. In Study 3, only 1 patient was treated with volasertib monotherapy; however, none of the AEs experienced by the patient were considered drug-related by the investigator.

The safety profile of volasertib in all studies was in line with previous studies [11, 12, 16]; the most common DRAEs, and all DLTs, were hematological in nature. Thrombocytopenia and neutropenia worsening was reported on treatment. However, as the cardinal symptoms of MDS patients are cytopenias, the symptoms of the underlying disease and the hematologic effects of volasertib (myelosuppression and cytopenias) are not distinguishable. Therefore, detection of the expected hematologic adverse effects of volasertib is challenging in MDS patients.

In Study 1, no MTD was determined up to a dose of 300 mg volasertib in combination with azacitidine. However, in Study 2, the dose of 200 mg volasertib in combination with azacitidine exceeded the MTD. This discrepancy might be explained by the difficulty to distinguish the symptoms of the underlying disease and the hematologic effects of treatment. Tolerability may have also been impacted by different patient characteristics (body weight, BSA) in Studies 1 and 2, due to a trend for a more pronounced myelosuppressive effect in patients with a lower weight.

To improve the tolerability of volasertib in combination with azacitidine in patients with MDS, BSA-based dosing and modified timing of administration to extend the treatment recovery period were investigated. However, the effect of these measures could not be assessed because the studies were terminated prematurely.

Preliminary signs of efficacy were observed in Studies 1 and 2, but these may have been due to the proven clinical activity of azacitidine alone, as documented in previous published studies [17–19]. Of note, most patients in Study 2 were previously treated with azacitidine; therefore, if these patients were refractory to azacitidine, the efficacy results may possibly be attributed to volasertib. Nevertheless, no firm conclusions on the clinical activity of volasertib plus azacitidine in MDS, CMML, or AML can be drawn from the small number of patients in these uncontrolled studies, especially as the efficacy assessments were not primary endpoints. Another limitation of the study is the absence of molecular data at baseline.

## Conclusions

Although these studies were halted for non-clinical reasons, the results presented in this article suggest that a tolerable dose of volasertib, administered in combination with azacitidine, could have been identified for the treatment of patients with MDS/CMML.

## Data Availability

To ensure independent interpretation of clinical study results and enable authors to fulfill their role and obligations under the ICMJE criteria, Boehringer Ingelheim grants all external authors access to relevant clinical study data. In adherence with the Boehringer Ingelheim Policy on Transparency and Publication of Clinical Study Data, scientific and medical researchers can request access to clinical study data after publication of the primary manuscript in a peer-reviewed journal, regulatory activities are complete and other criteria are met. Researchers should use the https://vivli.org/ link to request access to study data and visit https://www.mystudywindow.com/msw/datasharing for further information.

## Abbreviations

AE: Adverse event
AML: Acute myeloid leukemias
BSA: Body surface area
CMML: Chronic myelomonocytic leukemias
CR: Complete remission
CTCAE: Common Terminology Criteria for Adverse Events
D: Day
DLT: Dose-limiting toxicity
DRAE: Drug-related adverse event
GCP: Good Clinical Practice
HSCT: Hematological stem cell transplantation
LDAC: Low-dose cytarabine
MDS: Myelodysplastic syndromes
MCR: Marrow CR
MTD: Maximum tolerated doses
PD: Progressive disease
PLK: Polo-like kinases
PR: Partial remission
SAE: Serious adverse event
SD: Stable disease
